# Supplementary material for: Influenza A Virus Infection Reactivates Human Endogenous Retroviruses Associated with Modulation of Antiviral Immunity
Source: Viruses. 2022 Jul 21;14(7):1591. doi: 10.3390/v14071591 (PMC9320126; doi:10.3390/v14071591)
Supplement: Supplementary file 1 [file viruses-14-01591-s001.zip › MotifAnalysis.zip!/MotifOutput_SC35M/homerResults/motif10.similar.html]

motif10

## Information for motif10

A
G
T
C
C
G
A
T
C
G
T
A
A
C
T
G
A
T
G
C
A
C
G
T
G
T
C
A
C
G
T
A
A
C
T
G
A
C
T
G
  
Reverse Opposite:  

A
G
T
C
A
G
T
C
A
C
G
T
A
C
G
T
C
G
T
A
A
T
C
G
A
G
T
C
A
C
G
T
C
G
T
A
A
C
T
G
  

|  |  |
| --- | --- |
| p-value: | 1e-13 |
| log p-value: | -3.015e+01 |
| Information Content per bp: | 1.905 |
| Number of Target Sequences with motif | 9.0 |
| Percentage of Target Sequences with motif | 21.95% |
| Number of Background Sequences with motif | 188.8 |
| Percentage of Background Sequences with motif | 0.40% |
| Average Position of motif in Targets | 87.0 +/- 47.8bp |
| Average Position of motif in Background | 96.1 +/- 74.2bp |
| Strand Bias (log2 ratio + to - strand density) | 10.0 |
| Multiplicity (# of sites on avg that occur together) | 2.00 |
| Motif File: | file (matrix) reverse opposite |

### Similar de novo motifs found

|  |  |  |  |  |  |  |  |
| --- | --- | --- | --- | --- | --- | --- | --- |
| Rank | Match Score | Redundant Motif | P-value | log P-value | % of Targets | % of Background | Motif file |
| 1 | 0.716 | G A T C C T A G T A C G C G A T G T C A C G T A A C T G A C T G | 1e-9 | -21.520837 | 26.83% | 2.04% | motif file (matrix) |
